# Effects of Short-Term Low-Dose Glucocorticoids for Patients with Mild COVID-19

**DOI:** 10.1155/2020/2854186

**Published:** 2020-09-23

**Authors:** Hai-Yan Fu, Yu Luo, Jian-Peng Gao, Lin Wang, Hong-Juan Li, Xiang Li, Lian Xue, Xiao-Qing Tang, Hai-wen Li, Ying-Rong Du

**Affiliations:** ^1^Department of Palliative Care, The Sixth Affiliated Hospital of Dali University, The Third People's Hospital of Kunming, China; ^2^Department of Clinical Laboratory, The Sixth Affiliated Hospital of Dali University, The Third People's Hospital of Kunming, China; ^3^Department of Radiology, The Sixth Affiliated Hospital of Dali University, The Third People's Hospital of Kunming, China

## Abstract

**Objectives:**

To evaluate the role of short-term low-dose glucocorticoids in mild COVID-19 patients.

**Methods:**

We conducted a retrospective, cross-sectional, single-center study in Kunming, China. A total of 33 mild COVID-19 cases were divided into two treatment groups (with and without glucocorticoids, methylprednisolone, were used in this setting), and the absolute value of peripheral blood lymphocyte count; CD3+, CD4+, and CD8+ T cell counts; and the time to achieve negative transformation of a nucleic acid pharyngeal swab were recorded. Peripheral blood lymphocyte and T cell counts were compared between the treatment group and 25 healthy individuals. At the point of time when there was a 50% accumulation conversion rate (positive to negative nucleic acid on pharyngeal swab), and the nucleic acid turned negative in half of the patients in two groups, the peripheral blood lymphocyte and T cell counts were compared between treatment groups.

**Results:**

The mean cumulative time for the 50% negative conversion rate of the nucleic acid in the pharyngeal swab was 17.7 ± 5.1 days and 13.9 ± 5.4 days in the glucocorticoid group and the nonglucocorticoid group, respectively. The absolute peripheral blood lymphocyte count and the T cell subset count in the glucocorticoid group were lower than those in the nonglucocorticoid group. When the nucleic acid turned negative in half of the patients, the absolute value of peripheral blood lymphocyte count and CD4+ T cells of the glucocorticoid group and the nonglucocorticoid group was not significantly different; the CD3+ and CD8+ T cells in the glucocorticoid group were lower than those in the nonglucocorticoid group. The absolute peripheral blood lymphocyte count, CD3+ T cells, and CD4+ T cells in the glucocorticoid group were lower than those of the healthy group during the whole disease period, and CD8+ T cells returned to normal at 19-21 days of the disease period. There was no significant difference between the nonglucocorticoid group and the healthy group for absolute peripheral blood lymphocyte and CD8+ T cells; moreover, CD3+ T cells and CD4+ T cells were lower in the nonglucocorticoid group than those in the healthy group from the day of admission to the 18th day and returned to normal at the period of 19-21 days. The absolute peripheral lymphocyte count (*P* = 0.048, effect size *d* = 0.727) and T cell subset count (CD3: *P* = 0.042, effect size *d* = 0.655; CD4: *P* < 0.01, effect size *d* = 0.599; and CD8: *P* = 0.034, effect size *d* = 0.550) in the nonglucocorticoid group were higher than those in the glucocorticoid group, and the difference between the groups was statistically significant.

**Conclusions:**

This study found that the use of short-term, low-dose glucocorticoids does not negatively influence the clinical outcome, without affecting the final clearance of viral nucleic acid in mild COVID-19 patients.

## 1. Introduction

The coronavirus disease 2019 (COVID-19) has already become a global epidemic, and most infected patients have presented with mild symptoms. However, the burden of COVID-19 on the medical system has significantly increased due to the insidious disease progression after infection. Many patients presented with mild symptoms which rapidly progressed to severe condition and were associated with an increased risk of mortality [1]. It has remained unclear how to control the inflammatory response to prevent the aggravation of the disease in mild patients. Currently, the treatment strategies for COVID-19 include antiviral, antibiotic, immunoenhancement, and traditional Chinese medicine, while the role of glucocorticoids as an anti-inflammatory therapy for COVID-19 remains controversial [2–5]. Most existing evidence for the use of glucocorticoids in treating COVID-19 has come from China. According to the World Health Organization, the use of glucocorticoids in the clinical management for COVID-19 has been cautious, due to the fact that long-term high doses of glucocorticoids could cause immunosuppression and uncontrolled adverse events [6, 7]. However, there have been very few studies on the use of short-term low-dose glucocorticoids for patients with COVID-19. Therefore, a comprehensive investigation into the dynamic changes of immune function, the association between immune function and viral nucleic acid clearance, and effect of glucocorticoids on immune function is crucial for the treatment of mild COVID-19. This study is aimed at describing the changes in immune function and assessing the effect of short-term low-dose glucocorticoids on immune function and viral nucleic acid clearance for patients with mild COVID-19.

## 2. Methods

### 2.1. Patients

The protocol and amendments were approved by the ethics committee of the 3rd People's Hospital of Kunming (No.: 2020011513), all data collected were used for clinical study, and informed consent from the patient or guardian was obtained. The diagnosis criteria for COVID-19 were based on the “Diagnosis and treatment scheme of pneumonia infected by novel coronavirus” (sixth edition) issued by the National Health Commission and listed as follows [8]: (1) Epidemiology: travel or residence history in or around Wuhan, China, or other communities with reported cases within 14 days prior to symptom onset; a history of exposure to novel coronavirus infection (nucleic acid positive) within 14 days prior to symptom onset; exposure to someone with fever or respiratory symptoms from Wuhan City and surrounding areas, or from communities where cases were reported in the 14 days prior to the onset of illness; and exposure to a highly dense area of COVID-19 infections prior to the onset of illness. (2) Clinical manifestations: fever and/or respiratory symptoms and/or COVID-19 features present in chest imaging (in early-stage COVID-19, multiple small plaques and stromal changes are present, with obvious lung involvement. Disease progression then produces multiple ground-glass shadows and infiltrating shadows in both lungs. In severe cases, lung consolidation may occur, and pleural effusion is rare); and/or the number of white blood cells in the early stage of the disease was normal or decreased, and the lymphocyte count was decreased. Individuals that met at least 1 of the epidemiological criteria and 2 of the clinical manifestations, or with no criteria met in epidemiology but all 3 clinical manifestations, were diagnosed with COVID-19. Regardless of epidemiological or clinical manifestation, patients with 1 of the following etiological criteria were diagnosed with COVID-19: detection of novel coronavirus nucleic acid positive by real-time fluorescence RT-PCR, or virus gene sequencing was similar to the known novel coronavirus. The ordinary type was defined as follows: fever and/or respiratory symptoms, multiple small plaques and stromal change presence, and most ground-glass density with obvious lung involvement.

Contraindications for the use of glucocorticoids included [9] (1) unstable blood glucose control in diabetic patients; (2) allergy to methylprednisolone, hydrocortisone, dexamethasone, or other excipients; (3) refractory hypertension; (4) state of epilepsy or delirium; (5) glaucoma; (6) active gastrointestinal bleeding within the previous 3 months; or (7) known immunodeficient status. This study was a retrospective cohort study, and the patients who met the diagnostic criteria were included consecutively from those treated in the 3rd People's Hospital of Kunming from January 26, 2020, to March 2, 2020. The healthy control group comes from the physical examination center, and their physical examination conclusions were normal. Approved by the Ethics Committee of the 3rd People's Hospital of Kunming, the control group included in the study signed an informed consent form, authorizing the use of laboratory data on their blood routine and T cell subsets. A total of 33 patients with mild COVID-19 were included, and 25 healthy individuals were enrolled as the control group.

### 2.2. Clinical Data Collection

Patient data was collected including sex, age, location of residence, existing comorbidity, the absolute peripheral blood lymphocyte count during the entire disease period, T cell subset count, the sampling time of the penultimate pharynx swab before discharge (negative test), clinical type, and other indicators. Peripheral blood lymphocyte count and T cell subsets were collected for individuals in the normal control group from the physical examination center.

### 2.3. Treatment Strategy

All COVID-19 patients were examined with chest CT on the day of admission and reexamined on the fourth day after admission. Patients whose CT imaging showed evidence of mild increased pulmonary inflammation were given intravenous methylprednisolone 1 mg/kg/day as treatment on the fourth and fifth days. On the sixth day, if repeat CT was consistent with findings of nonprogressive pulmonary inflammation, the corticosteroids were discontinued after clinical evaluation of their condition and other clinical indicators. Other treatment strategies include lopinavir/ritonavir (200 mg, 2 tablets at a time, 2 times per day), with a course of 10 days or less based on the real-time COVID-19 diagnosis and treatment guidelines issued by the National Health Commission [8–10]. Other traditional treatment strategies included bed rest, oxygen therapy, and supportive treatment to ensure heat supply; maintaining water and electrolyte balance; and physical cooling which was applied if body temperature was less than 38.5°C and temporary oral antipyretic drugs which were used if body temperature is more than 38.5°C. Antibiotics were given according to imaging and inflammatory markers.

### 2.4. Statistical Analysis

Continuous variables were expressed as a mean ± SD, and the difference between groups was analyzed using a *t*-test. The statistical analysis was conducted using SPSS version 22.0 (IBM, Armonk, New York). The negative transformation of nucleic acid of the pharyngeal swab, the absolute value of lymphocyte count, and the T cell subgroup counts were graphed using Prism (GraphPad Prism 8; GraphPad Inc., San Diego, CA). All reported *P* values were two-sided, with a *P* value of <0.05 considered statistically significant.

## 3. Results

### 3.1. Base Characteristics

The general characteristics of the 33 mild COVID-19 patients enrolled in the study (15 male and 18 female) are summarized in Table 1.

### 3.2. The Time of Negative Nucleic Acid Test in Pharyngeal Swabs and the Changes of Lymphocyte Count and T Cell Subsets

**Figure 1:**
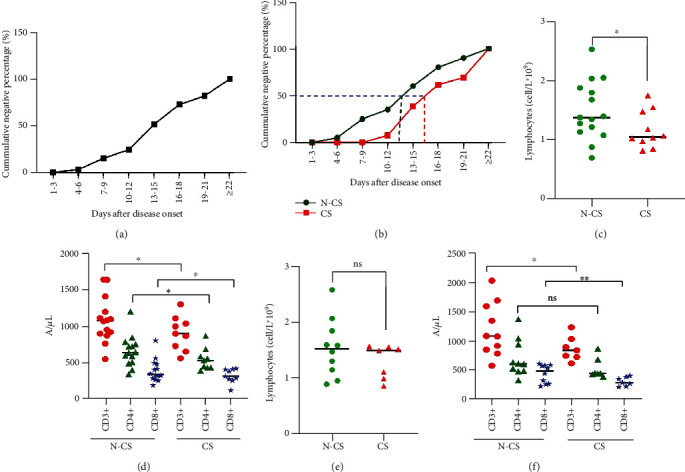
The nucleic acid conversion time and changes in T cells and lymphocytes in each group. (a) Cumulative negative conversion rate of nucleic acid at each time period in 33 cases. (b) Cumulative negative conversion rate of nucleic acid at each time period in the nonglucocorticoid group (N-CS) and the glucocorticoid group (CS), respectively. The 50% cumulative negative conversion rate (purple dashed line) in the nonglucocorticoid group (green dashed line) and the glucocorticoid group (red dashed line) after admission. (c) Distribution of lymphocytes in the nonglucocorticoid group (N-CS) and the glucocorticoid group (CS) when cumulative conversion reaches 50%. ^∗^*P* < 0.05. (d) Distribution of CD3+, CD4+, and CD8+ T cells in the nonglucocorticoid group (N-CS, 20 cases) and the glucocorticoid group (NS, 13 cases) when cumulative conversion reaches 50%. ^∗^*P* < 0.05. (e) The distribution of lymphocytes in half of the patients with the nonglucocorticoid group (N-CS, 10 cases) and the glucocorticoid group (NS, 7 cases). ns: not significant. (f) The distribution of CD3+, CD4+, and CD8+ T cells in half of the patients with the nonglucocorticoid group (N-CS, 10 cases) and the glucocorticoid group (CS, 7 cases). ^∗^*P* < 0.05; ns: not significant.

The mean time of the negative nucleic acid test in the pharyngeal swab was 15.5 ± 5.4 days, and the duration ranged from 6.0 to 28.0 days. The cumulative conversion rate of 50% was 13-15 days, and the cumulative negative conversion rate at each time period is shown in Figure 1(a). The nucleic acid conversion time for patients in the nonglucocorticoid group was 13.9 ± 5.4 days, while in the glucocorticoid group, this was 17.7 ± 5.1 days, and a significant difference was detected between the nonglucocorticoid and glucocorticoid groups; the *P* value was less than 0.05 (Supplementary Table 1). The 50% cumulative negative conversion rate for patients in the nonglucocorticoid group (purple dashed line in Figure 1(b)) occurred 13-15 days after admission (green dashed line in Figure 1(b)), and the 50% cumulative negative conversion rate for patients in the glucocorticoid group (purple dashed line in Figure 1(b)) occurred 16-18 days after admission (red dashed line in Figure 1(b)).

The cumulative negative conversion rate of 50% of COVID-19 patients for the nucleic acid pharyngeal swab test was different between the treatment groups. In addition, the absolute value of peripheral blood lymphocyte count and T cell subset counts also differs among the treatment groups. The cumulative negative conversion rate of 50% of patients treated with glucocorticoids was significantly longer than that in the nonglucocorticoid group. However, the absolute peripheral blood lymphocyte count (Figure 1(c)) and CD3+, CD4+, and CD8+ T cell count in the glucocorticoid group were lower than those in the nonglucocorticoid group (Figure 1(d)) (Supplementary Table 1).

When the nucleic acid turned negative in half of the patients, the absolute value of peripheral blood lymphocyte count (Figure 1(e)) and CD4+ T cells (Figure 1(f)) of the glucocorticoid group and the nonglucocorticoid group were not significantly different; the CD3+ and CD8+ T cells in the glucocorticoid group were lower than those in the nonglucocorticoid group; the *P* value was less than 0.05 (Figure 1(f)) (Supplementary Table 2).

### 3.3. Distribution of Peripheral Blood Lymphocyte Absolute Value and T Cell Subset Count

**Figure 2:**
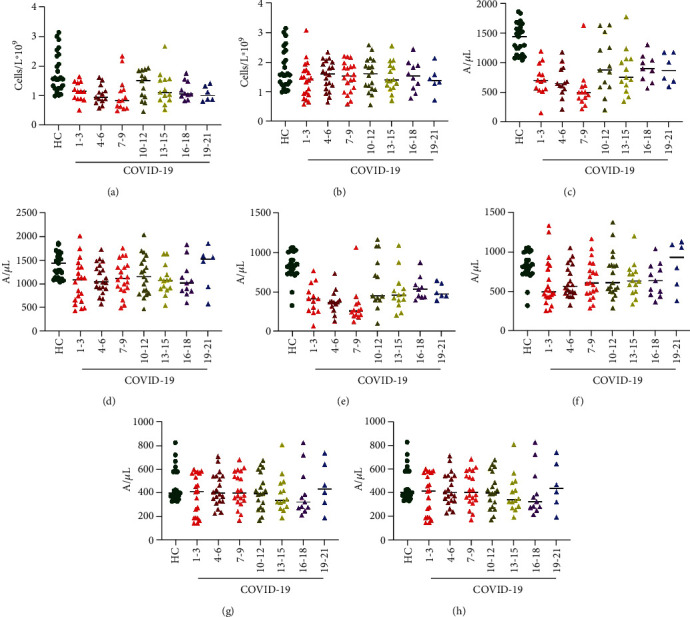
The distribution of lymphocytes in the healthy control group (HC) and COVID-19 patients at each period in (a) the glucocorticoid group and (b) the nonglucocorticoid group. The distribution of CD3+ T cells in the healthy control group (HC) and COVID-19 patients at each period in (c) the glucocorticoid group and (d) the nonglucocorticoid group. The distribution of CD4+ T cells in the healthy control group (HC) and COVID-19 patients at each period in (e) the glucocorticoid group and (f) the nonglucocorticoid group. The distribution of CD8+ T cells in the healthy control group (HC) and COVID-19 patients at each period in (g) the glucocorticoid group and (h) the nonglucocorticoid group.

The absolute value of peripheral blood lymphocyte count of COVID-19 patients in the glucocorticoid group was lower than that in the healthy control group during the hospitalization (Figure 2(a)) (Supplementary Table 3), and the level of peripheral blood lymphocytes was lowest at admission, then reached a peak after 10.0-12.0 days; the difference between groups was statistically significant. There was no statistically significant difference between the nonglucocorticoid group and the healthy control group for the absolute peripheral lymphocyte count (Figure 2(b)) (Supplementary Table 3). The CD3+ T cell count for COVID-19 patients in the glucocorticoid group was lower than that in the healthy control group during the hospital stay, with the lowest at 7-9 days and a peak at 10-12 days, and the difference was statistically significant (Figure 2(c)) (Supplementary Table 4). The absolute value of CD3+ T cell counts in COVID-19 patients in the nonglucocorticoid treatment group was lower than that in the healthy control group at the time of admission. As time progressed, CD3+ T cell count gradually rebounded until it returned to normal after 19.0 days (Figure 2(d)) (Supplementary Table 5). CD4+ T cell count for COVID-19 patients in the glucocorticoids group was lower than that in the healthy control group during hospitalization, with the lowest at 7-9 days and a peak at 10-12 days, with statistically significant differences (*P* < 0.05) (Figure 2(e)) (Supplementary Table 4). The fluctuation trend of CD4+ T cells was similar to that of CD3+ T cells. The absolute value of CD4+ T cells in the nonglucocorticoid group was lower than that of the healthy control group after admission and gradually increased with the extension of the disease period, until it returned to normal on the 19th day of admission (Figure 2(f)) (Supplementary Table 5). The change of CD8+ T cells was different from that of CD3+ T cells and CD4+ T cells. The CD8+ T cell count was lowest on days 7-9 and returned to normal on day 19 in the glucocorticoid group (Figure 2(g)) (Supplementary Table 4). In contrast, there was no statistically significant difference between the nonglucocorticoid treatment group and the healthy control group for CD8+ T cell count at admission, and it remained stable until discharge (Figure 2(h)) (Supplementary Table 5).

### 3.4. The Changes in Peripheral Blood Lymphocyte Counts and T Cell Subsets in the Glucocorticoid Group and the Nonglucocorticoid Group

**Figure 3:**
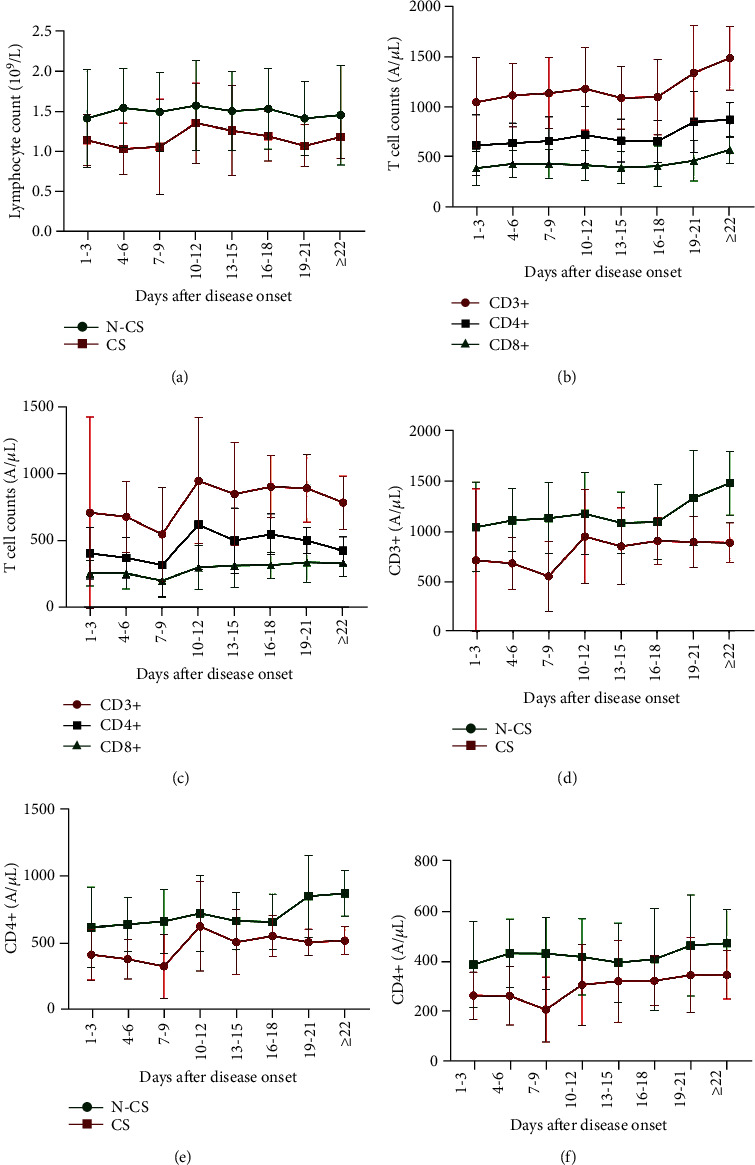
The change trend of the absolute value of peripheral blood lymphocytes and T cells. (a) The change trend of the absolute value of peripheral blood lymphocytes in the glucocorticoid group and the nonglucocorticoid group. The change trend of the CD3+, CD4+, and CD8+ T cells in (b) the nonglucocorticoid group and (c) the glucocorticoid group. The change trend of (d) CD3+ T cells, (e) CD4+ T cells, and (f) CD8+ T cells in the glucocorticoid group (CS) and the nonglucocorticoid group (N-CS).

The absolute peripheral blood lymphocyte count fluctuated between 1 and 1.5 × 109/L in both groups, and the absolute peripheral blood lymphocyte count in the nonglucocorticoid group (green line) was always higher than that in the glucocorticoid group (red line). In the nonglucocorticoid group, the peripheral blood lymphocyte count increased after 4.0 days and rose to a peak after 10.0-12.0 days and then remained stable until discharge. In contrast, in the glucocorticoid treatment group, the peripheral blood lymphocyte count was reduced after 4.0 days and rose to a peak after 10.0-12.0 days and then remained stable until discharge (Figure 3(a)).

For patients in the nonglucocorticoid group (Figure 3(b)), the trend of CD3+ and CD4+ T cell counts was similar, with counts gradually increased after admission, slightly decreased on days 13-15, plateaued on days 16-18, and then increased until discharge. CD8+ T cell counts in this group remained stationary throughout their treatment in the hospital (Figure 3(b)). For patients treated with glucocorticoids, the changes in CD3+, CD4+, and CD8+ T cell counts were similar, with all T cell counts reduced after the first treatment of glucocorticoids after 7.0-9.0 days. Closely following that point, CD3+, CD4+, and CD8+ T cell counts rapidly rose and reached a peak after 10.0-12.0 days, at which point they remained stable until discharge (Figure 3(c)).

The changes in CD3+, CD4+, and CD8+ T cell counts in the nonglucocorticoid and glucocorticoid treatment groups are presented in Figures 3(d)–3(f). The absolute value of CD3+, CD4+, and CD8+ T cell counts in the nonglucocorticoid group (green line) was higher than that in the glucocorticoid group (red line). At 7-9 days after admission (1 day after glucocorticoid use), there was a significant decrease in CD3+, CD4+, and CD8+ T cell counts. This increase in CD3+ (Figure 3(d)) and CD4+ (Figure 3(e)) T cell counts was statistically significantly different from that of the group not treated with glucocorticoids. The fluctuation of CD8+ T cells in the two groups was similar after 10.0-12.0 days, and both groups rose steadily until discharged (Figure 3(f)).

### 3.5. Clinical Outcome

33 patients who meet all the four criteria for discharge (mentioned below) were discharged [8]: (1) body temperature returned to normal for more than 3 days, (2) respiratory symptoms improved significantly, (3) lung imaging showed a significant improvement of inflammation, and (4) two consecutive (more than 24 hours) negative oropharyngeal swab nucleic acid tests.

## 4. Discussion

The treatment of COVID-19 has remained a difficult problem in the current worldwide pandemic, and the use of glucocorticoids during treatment has yielded inconsistent results. This study found that the use of short-term, low-dose glucocorticoids does not negatively influence the clinical outcome, without affecting the final clearance of viral nucleic acid.

Lymphocyte count and the T cell subgroup counts are decreased after infection by SARS-CoV-2 [11]. In the pathologic anatomic report of the first death due to COVID-19 in China, SARS-CoV-2 mainly attacked the lungs, and interstitial mononuclear inflammatory infiltration, dominated by lymphocytes, was seen in both lungs. This profile indicates a severe injury due to immune response in patients with COVID-19 [12]. The findings of the current study were consistent with this previous autopsy report, despite the fact that patients recruited in this study displayed mild symptoms. This result suggested that lymphocytes and CD8+ T cells may be important in monitoring disease progression, which is consistent with the study conducted by Chan et al. [13]. In this study, the use of a low dose of glucocorticoids as treatment was based on the inflammatory progression noted in CT imaging.

Immune function, as the sentinel of the body, is an important factor in the clearance of viral infection. Interestingly, the negative time of nucleic acid required for peripheral blood lymphocytes and T cell subgroup counts between treatment groups are significantly different. In addition, elimination of the virus was also completed when the levels of peripheral blood lymphocytes and CD3+, CD4+, and CD8+ T cell counts were significantly lower than patients in the nonglucocorticoid group. These results suggested that several other factors other than normal immune function were involved in virus clearance in patients with COVID-19, and the reduced inflammation response might accelerate virus clearance.

It is worth noting that the level of T cell subsets in the nonglucocorticoid group was higher than that in the glucocorticoid group at the time of admission, so the delayed viral clearance in the glucocorticoid group is not necessarily caused by the use of glucocorticoids. But this study was retrospective, and allocation to treatment was not randomized; therefore, clinical status may be partly responsible for the differences between two groups. A recent RECOVERY trial from Oxford University showed that dexamethasone could lower 28-day mortality in patients hospitalized with COVID-19 [14]. Both our research and the Oxford University research have confirmed the benefit of glucocorticoids in COVID-19 to some extent. In SARS-CoV patients, early high-dose glucocorticoids can produce the most favorable outcomes [15], and for other infectious diseases, glucocorticoids were also recognized to be clinically effective and safe [16]. So, if not contraindicated, glucocorticoids should be considered in SARS, MERS, and COVID-19 patients [17]. There were different opinions about using glucocorticoids to treat COVID-19 patients. A recent meta-analysis about glucocorticoid therapy in COVID-19 showed that glucocorticoid can reduce the duration of fever, but long-term high-dose glucocorticoids increased the risk of coinfections or osteoporosis, so systemic glucocorticoid therapy cannot be recommended, but in clinical trials, short-term low-dose glucocorticoid may be acceptable [18].

The T cell subgroup counts showed a temporary decline after the short-term, low-dose treatment of glucocorticoids. This was followed by a significant increase in the level of T cell count to peak levels, a trend that was similar between treatment groups at the end stage of disease progression. One previous study has already found that the use of glucocorticoids in the first 3.0 days after admission could prevent further deterioration for patients with severe COVID-19 [19]. Moreover, the use of short-term, low-dose (methylprednisolone 0.5 mg–1 mg per kg per day for 5 days) glucocorticoids could prevent potential specific adverse events associated with prolonged glucocorticoid use [19]. In this study, there were no specific adverse events related to glucocorticoid use.

Glucocorticoids played an important role in the treatment of fever, pulmonary edema, the formation of a transparent membrane of the lung, and the improvement of blood oxygen saturation infected by SARS-CoV-2 [20–22]. However, several adverse events, including opportunistic infections, ventilator-associated infections, and increased plasma viral load in non-ICU patients with SARS, were found in patients after the use of glucocorticoids [20]. Russell et al. [23] summarized the use of glucocorticoids in SARS-CoV-2 and MERS-CoV studies and concluded that glucocorticoids were not suitable for patients with COVID-19. The potential reason for this included diabetes, psychosis, and delayed virus clearance. These adverse events and immunosuppression are typically detected after the use of long-term, high dosages of glucocorticoids, and a study has already demonstrated that the average peak dose and mean treatment duration are significantly correlated with osteonecrosis [24]. A study conducted by Zhang et al. found that a low dose of dexamethasone could alleviate the early inflammatory response to coronavirus infection in the acute stage of infection, while the use of long-term dexamethasone could enhance viral replication [25]. Similar results were also detected using mouse models, pointing to an association between the inflammation and lung tissue damage caused by SARS-CoV-2 and the increase and imbalance of proinflammatory and anti-inflammatory cytokines [26]. In our study, all of the patients treated with glucocorticoids remained in the mild stage of COVID-19 and did not progress to the severe or critical stage, and complete virus clearance was achieved in all cases. Moreover, there was no virus reactivation in either group after 14.0 days of follow-up.

In conclusion, changes in immune function should be closely monitored for patients with mild COVID-19. The use of short-term, low-dose glucocorticoids could offer additional benefits for patients with mild COVID-19 without affecting the final clearance of viral nucleic acid.

## Figures and Tables

**Table 1:** General characteristics of patients with COVID-19.

Variables	Number of patients	Proportion (%)
Sex		
Male	15	45.0
Female	18	55.0
Age		
0-6 years	0	0
7-17 years	4	12.1
18-40 years	6	18.2
41-65 years	18	54.6
≥66 years	5	15.6
Comorbidity		
Hypertension	5	15.2
Diabetes	2	6.1
Clinical classification		
Mild	3	9.1
Ordinary	30	90.9
Residence		
Hubei	18	55.0
Yunnan	15	45.0
Lymphocyte absolute value after admission (normal: 0.8‐4 × 10^9^/L)		
Normal	28	84.9
Reduced	5	15.2
CD3+ T cell after admission (normal: 1027-2086/*μ*L)		
Normal	20	60.6
Reduced	13	39.4
CD4+ T cell after admission (normal: 706-1125/*μ*L)		
Increased	2	6.1
Normal	5	15.2
Reduced	26	78.8
CD8+ T cell after admission (normal: 323-836/*μ*L)		
Normal	16	48.5
Reduced	17	51.52
Treated with glucocorticoids		
Yes	13	39.4
No	20	60.6

## Data Availability

The original data used to support the findings of this study are restricted by the ethics committee of the 3rd People's Hospital of Kunming in order to protect patient privacy. Data are available from Ying-Rong Du (contact emails: dyr_km@163.com) for researchers who meet the criteria for access to confidential data.

## Supplementary Materials

Supplementary Materials Table 1: comparison of peripheral blood lymphocyte count and T cell subgroup count with a cumulative negative conversion rate of 50% in the pharyngeal swab. Table 2: comparison of peripheral blood lymphocyte count and T cell subgroup count when 50% of patients had a negative nucleic acid test in the pharyngeal swab. Table 3: changes in the absolute number of peripheral blood lymphocytes in the glucocorticoid group and the nonglucocorticoid group (10^9^/L). Table 4: changes in peripheral blood T cell subset count in COVID-19 patients treated with glucocorticoids. Table 5: changes in peripheral blood T cell subset count in patients without glucocorticoids.
