# Supplementary material for: Effects of Short-Term Low-Dose Glucocorticoids for Patients with Mild COVID-19
Source: Biomed Res Int. 2020 Sep 23;2020:2854186. doi: 10.1155/2020/2854186 (PMC7519460; doi:10.1155/2020/2854186)
Supplement: Supplementary Materials — Table 1: comparison of peripheral blood lymphocyte count and T cell subgroup count with a cumulative negative conversion rate of 50% in the pharyngeal swab. Table 2: comparison of peripheral blood lymphocyte count and T cell subgroup count when 50% of patients had a negative nucleic acid test in the pharyngeal swab. Table 3: changes in the absolute number of peripheral blood lymphocytes in the glucocorticoid group and the nonglucocorticoid group (109/L). Table 4: changes in peripheral blood T cell subset count in COVID-19 patients treated with glucocorticoids. Table 5: changes in peripheral blood T cell subset count in patients without glucocorticoids. [file 2854186.f1.docx]

**Table 1. Comparison of peripheral blood lymphocyte count and T cell subgroup count when cumulative negative conversion rate of 50% in pharyngeal swab**

| Treatment group | Glucocorticoids group (n=13) | Non-glucocorticoids group (n=20) |
| --- | --- | --- |
| Time since onset (days) | 17.7±5.1 | 13.9±5.4* |
| Absolute value of peripheral blood lymphocytes count（10^9^/L) | 1.2±0.3 | 1.5±0.5* |
| CD3+ T cell count (/ul) | 906.2±233.4 | 1085.36±308.4* |
| CD4+ T cell count (/ul) | 550.2±151.2 | 660.8±212.7* |
| CD8+ T cell count (/ul) | 321.4±98.2 | 393.3±156.3* |

***compared with patients treated with glucocorticoids: *p*<0.05**

**Table 2. Comparison of peripheral blood lymphocyte count and T cell subgroup count when 50% of patients had a negative nucleic acid test in pharyngeal swab**

| Treatment group | Glucocorticoids group (n=7) | Non-glucocorticoids group (n=10) |
| --- | --- | --- |
| Absolute value of peripheral blood lymphocytes count（10^9^/L) | 1.3±0.3 | 1.5±0.5 |
| CD3+ T cell count (/ul) | 873.9±208.3 | 1200.3±458.9* |
| CD4+ T cell count (/ul) | 535.1±176.3 | 707.4±318.5 |
| CD8+ T cell count (/ul) | 310.1±76.5 | 444.9±156.9* |

***compared with patients treated with glucocorticoids: *p*<0.05**

**Table 3. Changes in the absolute number of peripheral blood lymphocytes in glucocorticoids group and non-glucocorticoids group (10^9^/L)**

| Patient Group | 1-3 days | 4-6 days | 7-9 days | 10-12 days | 13-15 days | 16-18 days | 19-21 days | ≥22 days | Healthy control |
| --- | --- | --- | --- | --- | --- | --- | --- | --- | --- |
| Glucocorticoids group | 1.1±0.3* | 1.0±0.3* | 1.1±0.6* | 1.4±0.5* | 1.3±0.6* | 1.2±0.3* | 1.1±0.3* | 1.2±0.3* | 1.7±0.6 |
| Non-glucocorticoids group | 1.4±0.6 | 1.5±0.5 | 1.5±0.5 | 1.6±0.6 | 1.5±0.5 | 1.5±0.5 | 1.4±0.5 | 1.4±0.5 |  |

***significantly different from healthy control: p<0.05**

**Table 4. Changes in peripheral blood T cell subsets count in COVID-19 patients treated with glucocorticoids**

|  | 1-3 days | 4-6 days | 7-9 days | 10-12 days | 13-15 days | 16-18 days | 19-21 days | ≥22 days | Healthy control |
| --- | --- | --- | --- | --- | --- | --- | --- | --- | --- |
| CD3+ | 711.1±268.3* | 680.8±262.1* | 550.4±350.9* | 949.7±468.4* | 851.2±384.6* | 906.2±233.4* | 894.3±253.8 | 787.0±191.46* | 1400.8±255.0 |
| CD4+ | 410.2±190.1* | 377.2±148.7* | 323.3±236.2* | 622.2±334.1* | 503.8±242.3* | 550.2±151.2* | 504.8±98.2* | 427.67±104.14* | 830.3±173.0 |
| CD8+ | 262.2±94.0* | 261.2±115.5* | 207.6±128.4* | 305.1±160.2* | 319.4±162.5* | 321.4±98.2* | 343.7±149 | 338±96.42 | 462.3±137.7 |

***significantly different from healthy control: p<0.05**

**Table 5. Changes in peripheral blood T cell subsets count in patients without glucocorticoids**

|  | 1-3 days | 4-6 days | 7-9 days | 10-12 days | 13-15 days | 16-18 days | 19-21 days | ≥22 days | Healthy control |
| --- | --- | --- | --- | --- | --- | --- | --- | --- | --- |
| CD3+ | 1043.1±446.9* | 1111.1±312.5* | 1132.4±351.9* | 1177.6±410.6* | 1085.36±308.4* | 1094.1±374.5* | 1334±473.1 | 1480.0±316.2 | 1400.8±255.0 |
| CD4+ | 613.8±302.1* | 635.3±202.3* | 658.2±238.8* | 718.2±282.3* | 660.8±212.7* | 654.5±208.2* | 847±304.8 | 868.0±170.0 | 830.3±173.0 |
| CD8+ | 385±170.9 | 429.9±135.9 | 429.1±143.6 | 415.5±150.9 | 393.3±156.3 | 406.1±200.8 | 460.7±200.3 | 571.0±134.59 | 462.3±137.7 |

***compared with healthy control: p<0.05**
